# Remedies for Cough

**Published:** 1877-06-20

**Authors:** 


### REMEDIES FOR COUGH.

We may profitably study a few of the remedies for cough in this place, though most of them have been named in the treatment of diseases of the respiratory apparatus. But as we well know, it requires frequent repetition to impress facts upon our minds. Let us think of the two-fold treatment—"remedies that remove the disease will remove the cough, and remedies that remove the cough favorably influence the disease." The first is especially applicable to acute, and the second to chronic diseases. If, for instance, we find a cough associated with increased temperature, a frequent pulse, arrest of secretion, and impairment of innervation, we conclude at once that remedies which will rectify these wrongs will relieve the cough. One might think that this was a very natural conclusion, and yet we find that many practitioners do not reach it, but commence with special remedies called expectorants.

In studying the old group of *expectorants* they were divided into two prominent classes—nauseant, or relaxant, and stimulant. If there was arrest of secretion, the first class would furnish the remedies; if there was profuse secretion, they would be drawn from the second group. Once in a while we go back to these old remedies and select them in this way. But the trouble in their use is that they do too much. If there is dryness of the respiratory passages, and we give the "nauseant expectorants," we are pretty sure to get a too abundant secretion; so, in some cases, the stimulants do too much, and arrest the secretion, or make it difficult of removal.

Let me record it that *expectoration* is morbid, not natural; that a profuse secretion of mucus, or muco-pus, is not essential to recovery, but is produced by impairment of the life of the part, and leads to further impairment.

*Lobelia.*—I prefer to use lobelia for its stimulant action, rather than as a nauseant expectorant. If there is a sense of oppression and feeling of dullness, I should prescribe this remedy without reference to the arrest of secretion. As we have already seen, it is the remedy in asthenic bronchitis, especially of children, with profuse secretion and difficulty in removing the accumulations. Let me repeat the formula for the child: R. Tinct. lobelia (seed) dr. j, comp. tinct. lavender dr. iij, simple syrup oz. iss. M.

*Ipecacuanha.*—Giving up the old use of ipecac as a nauseant, let us employ it as a remedy for that irritation which prompts an almost continued effort to free the larynx by cough. It may be a case of inflammation, as in infantile pneumonia, or it may be only a cough, but with this symptom the remedy is very effectual. It may be triturated with sugar, or sugar and gum arabic and given in small doses, or the tincture may be prescribed in water.

*Sanguinaria.*—I prefer the nitrate of sanguinaria to any other preparation, as its action seems better, and it is more easily carried and dispensed. One grain to four ounces of simple syrup makes a very good preparation, and when dispensing from my pocket case I add it to water in the same proportion. The indication, for it is a sense of constriction and tickling in the throat.

*Tartar Emetic.*—But you don't use any antimony, do you? O no, I get along very well without it, but should you wish to use it, I will name the cases and the quantity. If there is hoarseness with tenderness of the larynx, you have a marked case; or if the cough is hollow and reverberating, and there is evidently want of power in it, you have the other. Triturate the remedy with sugar, one grain to one drachm, and divide in forty powders, giving one in every two or three hours. You could hardly kill any one with this, though, if a child, we would make the dose still smaller.

*Belladonna.*—Belladonna is a cough medicine as well as a remedy for congestion, as we have already seen whilst studying whooping cough. The indication is the usual one—dullness and inclination to sleep.

*Drosera.*—Drosera is the remedy for the cough of measles, and for any cough that simulates to it. This cough, it will be recollected, is paroxysmal and explosive. The proportions are—R. Tinct. drosera dr. ss to dr. j, water oz. iv; a teaspoonful every four hours.

*Nitric Acid.*—Nitric acid is frequently a most admirable cough remedy, and will quiet irritation when others fail. If now we should stop there, how would one know when to use it? But if we say whenever the tongue shows the marked *violet* color, we use nitric acid, any one can select the case for the remedy.

*Stillingia.*—This is a most valuable remedy for the relief of cough, and for some of the most intractable forms. The irritation seems to localize itself just back of the posterior pillars of the fauces, and sometimes above the soft palate, though there may be disease of any part of the respiratory apparatus. In ordinary prescribing the tincture of stillingia may be added to any cough mixture; or it may be prescribed with simple syrup; or it may be dispensed in water like other remedies. We have already called attention to the stillingia liniment as a most valuable remedy.

*Sticta Pulmonaria.*—This is a most valuable remedy, and if the characteristic indications present, it will speedily check a cough. There is pain in the shoulders, passing up through the neck to the occiput; the cough is frequently violent, and so harassing as to prevent rest. R. Tinct. sticta gtt. x to dr. ss, to water oz. iv; a teaspoonful every two or three hours.

*Macrotys.*—With pain, evidently in the thoracic walls, rheumatic in character, I use macrotys as a cough remedy.

*Collinsonia.*—We have found that collinsonia was a remedy for irritable larynx, ministers' sore throat, and a chronic laryngitis. But we have diseases of other parts of the respiratory apparatus in which the use of the voice brings on cough. Let us try the collinsonia in these cases.

*Grindelia.*—We will think of this as a possible remedy for asthmatic cough, and as a pretty certain remedy for a chronic cough associated with profuse leucorrhœa, or with a discharge of mucus or muco-pus with triple phosphates in the urine. This is rather a singular indication for a cough medicine, but it will be well to note it. The dose will vary from one to ten drops.

*Bromide of Ammonium.*—In the study of whooping cough we found that this was the remedy for an epileptiform cough, and in treating children I would suggest its use when the slightest involuntary movement of muscles is observed.

*Cactus.*—Cactus is a remedy for cough when associated with præcordial oppression and irregularity of pulse.

*Pulsatilla.*—Dizziness, fear of impending danger, and the condition of the mind designated as nervousness, suggest this remedy.

This only names a few of the remedies that will influence a cough, but it will suggest the method of selecting the right remedy. The rule in all cases is, "give the remedy called for by characteristic symptoms," and in this way we can hardly make a mistake.

*Remedies that influence the Fauces.*—For the temporary relief of cough we frequently employ remedies in such form that they influence the fauces or throat, and find it much better than their internal administration. The simplest way is to use a lump of sugar, and drop the remedy upon it as has been named for the stillingia liniment. Equal parts of sugar and gum arabic form a very good basis for the administration of remedies, and will sometimes quiet a cough themselves by their demulcent action. The powder is allowed to dissolve slowly in the mouth, and then swallowed. Any thing that may be thought useful may be added, as chlorate of potash, alum, borax, aconite, morphine, lobelia, stillingia, etc.

*Remedies by Inhalation.*—Remedies by inhalation exert a direct influence upon the respiratory mucous membrane, and are sometimes used with great advantage. Volatile agents may be inhaled from the old-fashioned glass inhaler, or from a glass tube in which a loose sponge has been placed, the remedy being dropped upon this. The latter method is a very good one. Some of the remedies are burned and the gas or smoke is inhaled, as is the case with the many "burners" used for asthma. Others being more volatile are used with the spray apparatus, or atomizer, and the finely divided fluid is carried into the lungs with the respired air. It is not necessary to give formulæ for these here.—*Eclec. Med Jour.*

